# Supplementary material for: Design principles for enhancing phase sensitivity and suppressing phase fluctuations simultaneously in biochemical oscillatory systems
Source: Nat Commun. 2018 Apr 12;9:1434. doi: 10.1038/s41467-018-03826-4 (PMC5897384; doi:10.1038/s41467-018-03826-4)
Supplement: Supplementary file 1 — Supplementary Information [file 41467_2018_3826_MOESM1_ESM.pdf]

Supplemental Information

Supplementary Figures

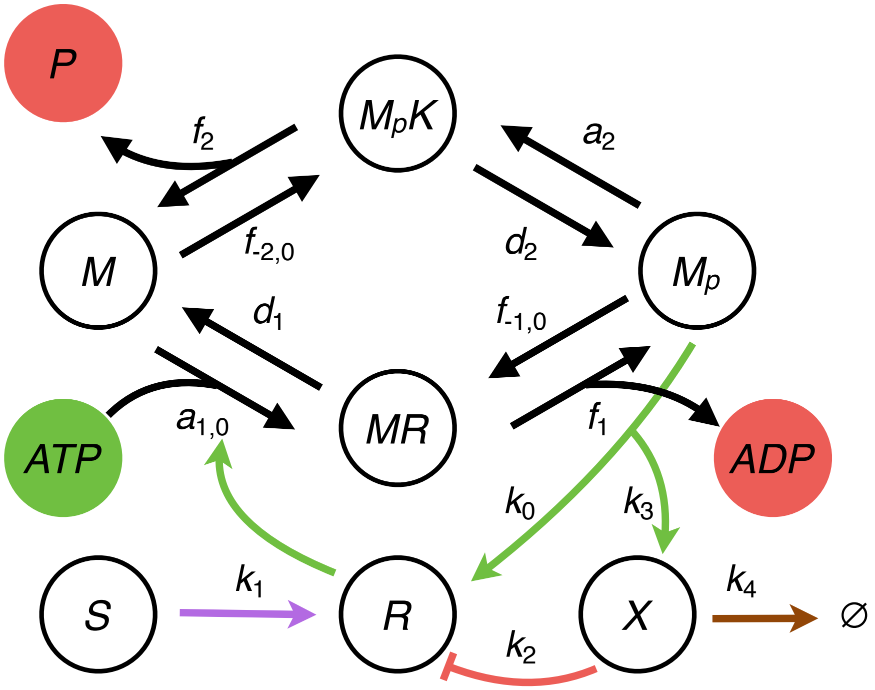

Supplementary Figure 1: Illustration of AI model. We include ATP, ADP and P explicitly in the PdP cycle. Controlling ATP and ADP/P_i concentration is equivalent to regulate forward/backward reaction rates through γ_1 and γ_2. We choose $a_{1,0}[\mathrm{ATP}]^{(\mathrm{eq})}$=0.01, $f_{-1,0}[\mathrm{ADP}]^{(\mathrm{eq})}$=$f_{-2,0}[\mathrm{P}_i]^{(\mathrm{eq})}$=1 and set [ADP]=[$P_i$] so that there is only two free parameters. We introduce γ_1=$[\mathrm{ATP}]^{(\mathrm{eq})}/[\mathrm{ATP}]$ and γ_2=$([\mathrm{ADP}]\cdot[\mathrm{P}_i])/([\mathrm{ADP}]^{(\mathrm{eq})}\cdot[\mathrm{P}]^{(\mathrm{eq})})$. The equilibrium state is restricted by $\gamma_1\gamma_2 = 1$.

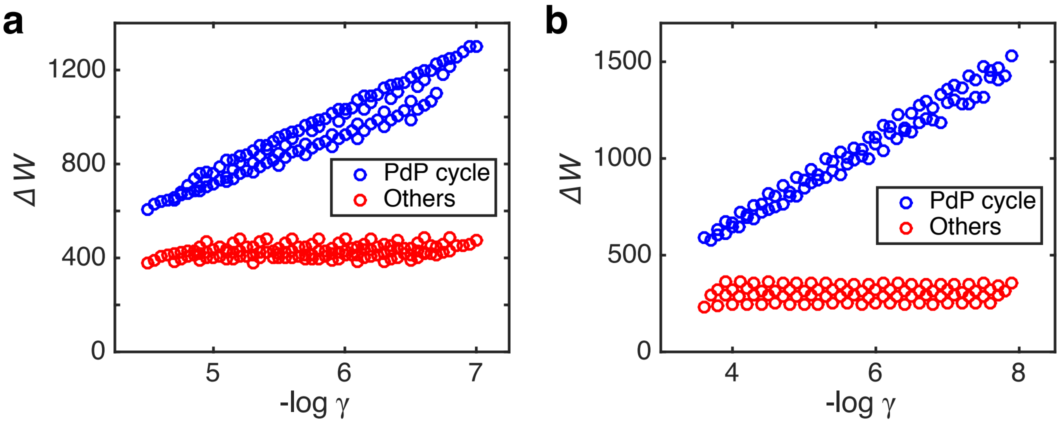

Supplementary Figure 2: Irreversibility affects oscillation property by controlling the free energy dissipated in the PdP cycle. ΔW is computed for both **(a)** the direct reverse scheme, and **(b)** the Michaelis-Menten scheme in the full AI model. Dissipation from the PdP cycle (blue) and other reactions outside the PdP cycle (red) are calculated separately. The part of dissipation that increase significantly when varying γ comes from the PdP cycle while the other contributions from outside the PdP cycle remains roughly constant.

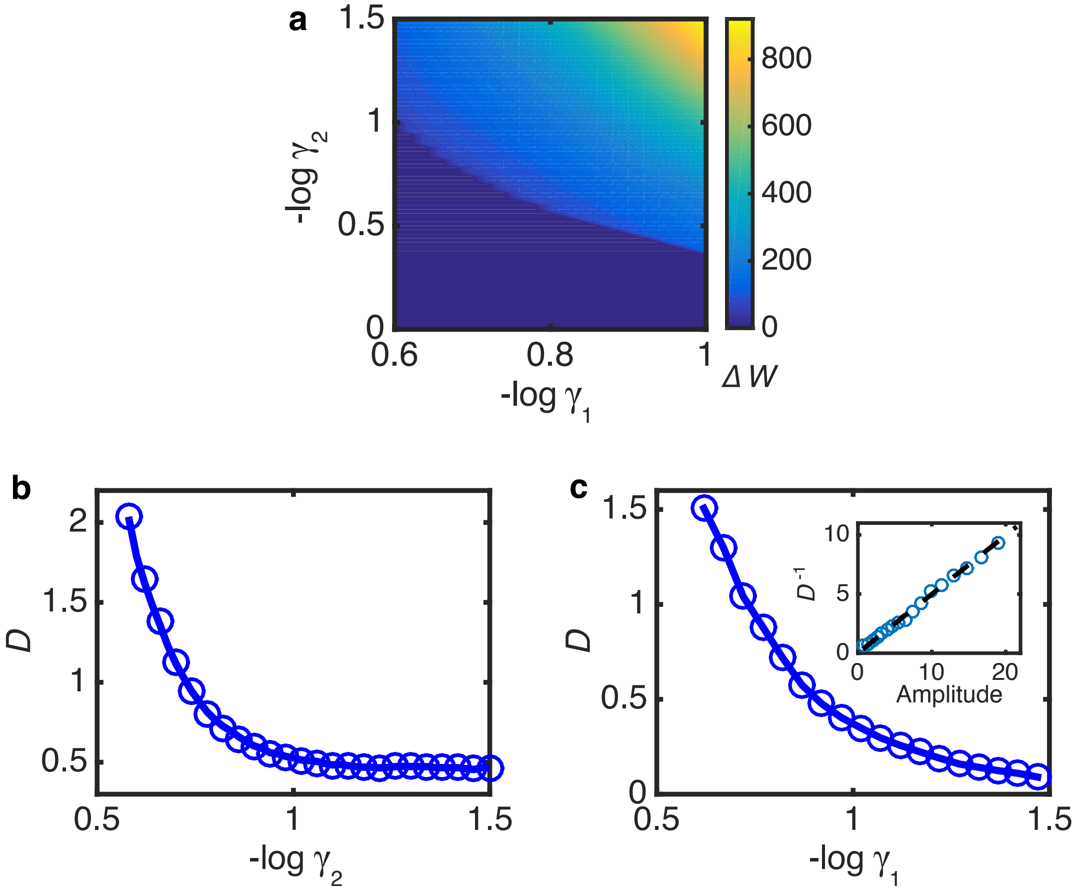

Supplementary Figure 3: Different strategies of spending free energy decrease phase diffusion differently. **(a)** Dependence of energy dissipation per period ΔW on irreversibility parameter γ_1 and γ_2. **(b)** $\gamma_1 = 0.8$. D decreases with $1/\gamma_2$ and eventually saturates to a nonzero value. **(c)** $\gamma_2 = 1$. D continues to decrease as γ_1 decrease. The relative period variance declines as one over the oscillation amplitude, indicating the $1/N$ averaging effect.

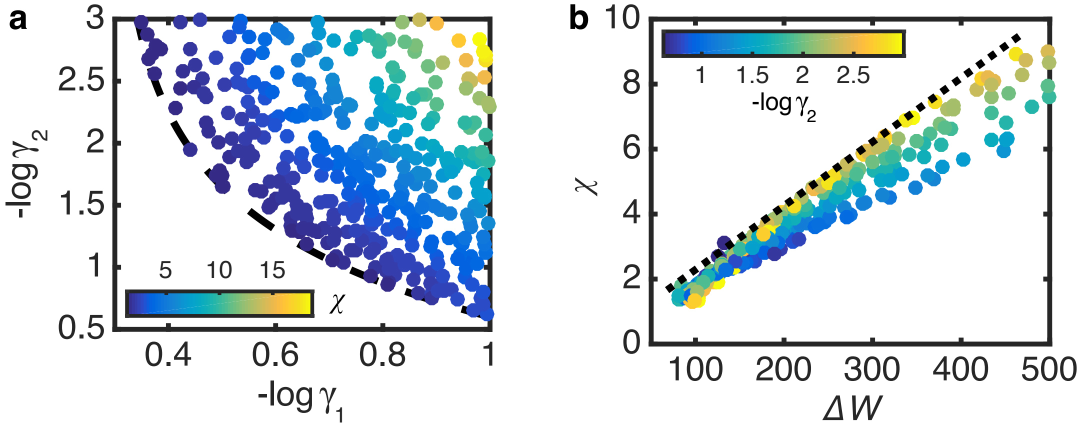

Supplementary Figure 4: Testing a different way of energy allocation in Brusselator. Simulation parameters are: $k_2 = k_2^{(\mathrm{eq})}/\gamma_1$, $k_{-2} = k_{-2}^{(\mathrm{eq})}/\sqrt{\gamma_2}$, and $k_{-3} = k_{-3}^{(\mathrm{eq})}/\sqrt{\gamma_2}$. **(a)** Different combinations of γ_1 and γ_2 are randomly chosen above the bifurcation (dashed line). It is clear that phase sensitivity χ grows when farther away from thermal equilibrium. **(b)** The sensitivity-energy relation still indicates that χ is linearly enhanced by dissipation per cycle. The dashed line is fitted by $\chi_{\max} = K_W \Delta W + \text{const.}$. The fitting parameters is $K_W = 2.00 \times 10^{-2}$.

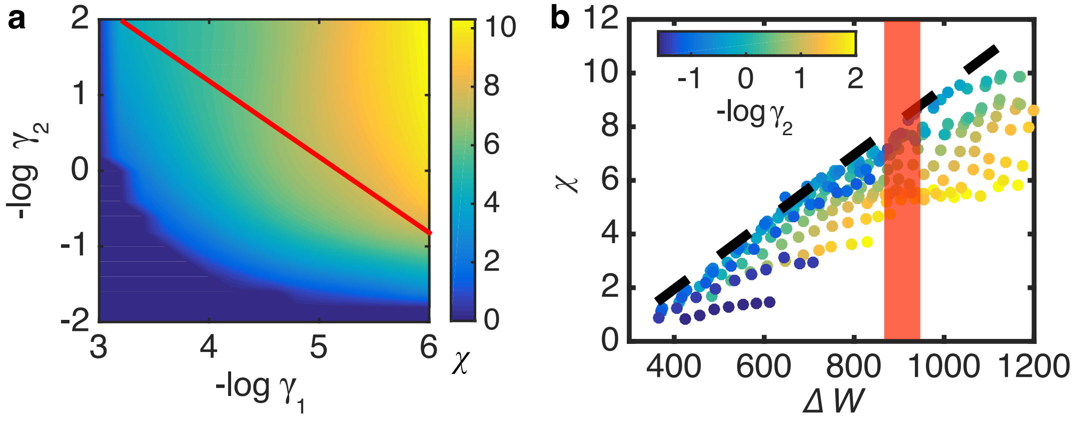

Supplementary Figure 5: Free energy enhances phase sensitivity in AI model. In living organisms, the free energy released by ATP hydrolysis is typically $\sim 12 k_B T$, from which we infer that $\gamma = \gamma_1 \gamma_2 \approx 10^{-5.2}$. This value is shown as the red lines. **(a)** Dependence of phase sensitivity χ on parameter γ_1 and γ_2 in AI model. **(b)** The dependence of phase sensitivity χ on energy dissipation per period ΔW for different choices of $\gamma_{1,2}$. The linear relationship between the upper limit of χ and the energy dissipation ΔW is shown by the black dashed line, and is fitted by $\chi_{\max} = K_W \Delta W + \text{const.}$ with the linearity parameter $K_W \approx 1.26 \times 10^{-2}$. The width of the red line shows the range of ΔW for a realistic value of $\gamma = 10^{-5.2}$.

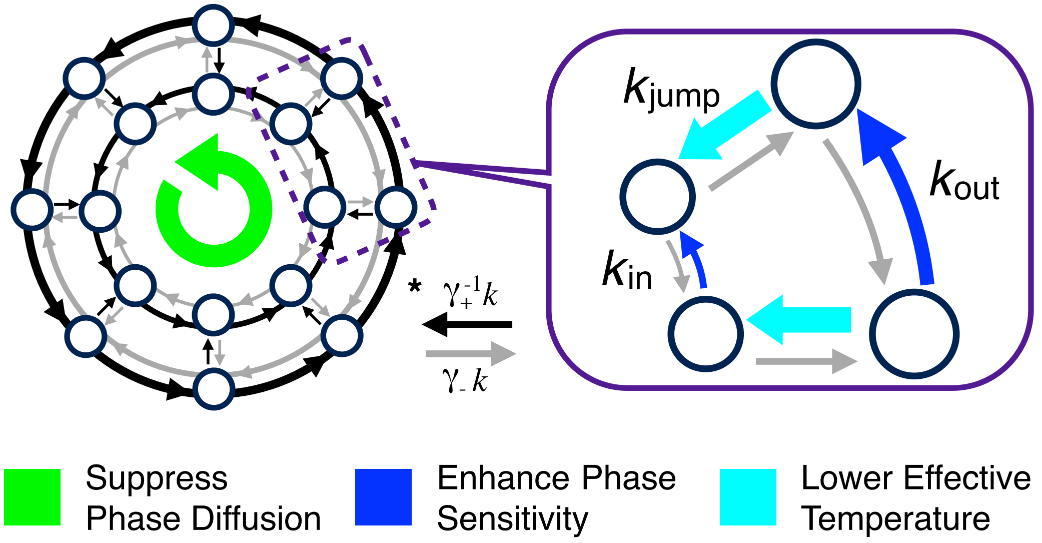

Supplementary Figure 6: A toy model for an ideal limit cycle oscillator. Two adjacent circles with N states each are considered. The inner cycle is the stable limit cycle. The outer cycle represents the perturbed states where the phase velocity is different from that on the limit cycle. The equilibrium transition rates between adjacent states (both forward and backward) are given by k_{in}, k_{out}, and k_{jump} shown. All the rates are regulated by nonequilibrium parameters γ_+ (enhancing forward) and γ_- (suppressing backward) the same way. Three essential roles of free energy cost in regulating oscillatory behaviors are illustrated at the bottom.

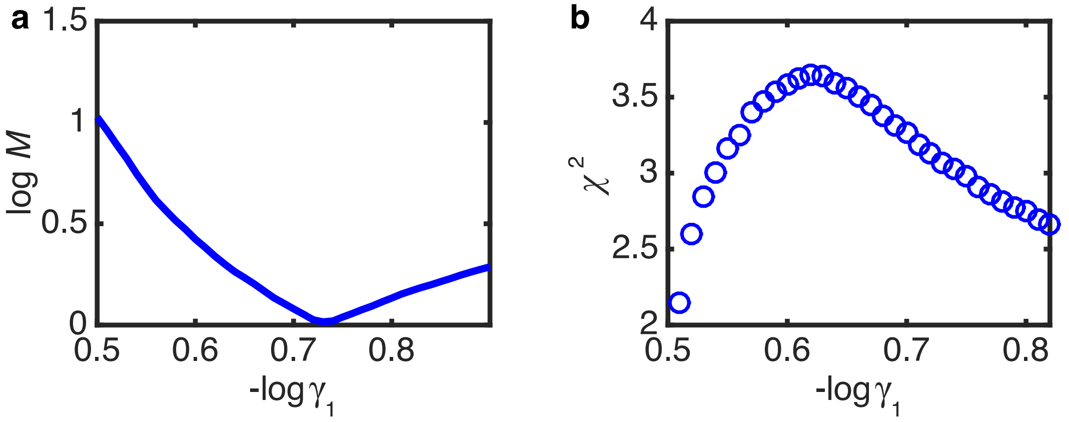

Supplementary Figure 7: Optimal oscillation functions of Brusselator given a fixed energy budget. While changing different combinations of γ_1 and γ_2 with fixed dissipation $\Delta W = 100$, there exists **(a)** minimum phase diffusion and **(b)** maximum phase sensitivity. In this case, the ideal performance can be achieved at $-\log\gamma_1 \approx 0.65$.

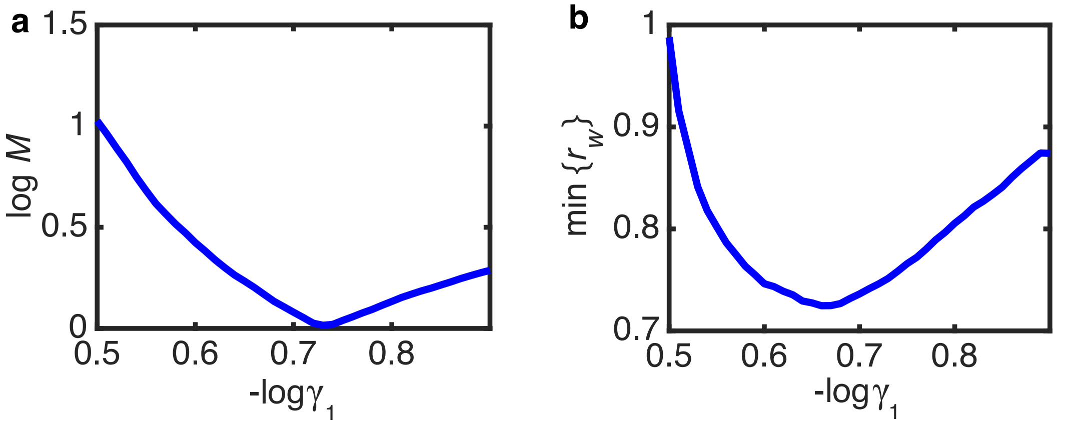

Supplementary Figure 8: Best strategy to utilize free energy for both low fluctuation and high sensitivity. ΔW is fixed at 100 in Brusselator model. **(a)** M is plotted against $-\log\gamma_1$. The minimum of phase diffusion is reached when the forward and backward flux ratios are matched among different reaction pathways. **(b)** The dependence of net flux ratio r_w on different combinations of γ_1 and γ_2 suggests a negative correlation with phase sensitivity.

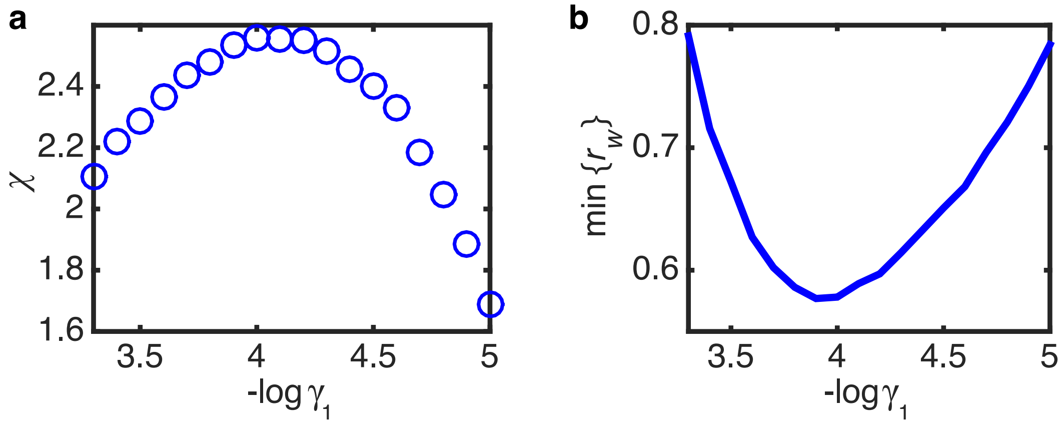

Supplementary Figure 9: Design principle for phase responsiveness in AI model. We varied $\gamma_{1,2}$ and fixed $\Delta W = 500$. **(a)** χ is plotted against $-\log\gamma_1$. Phase sensitivity peaks at $-\log\gamma_1 \approx 4$. **(b)** In AI model, we denote $w_1 = f_{-2}[M][K]-f_2[M_pK], w_2 = d_2[M_pK]-a_2[M_p][K], w_3 = f_{-1}[M_p]([R]-[MR])-f_1[MR], w_4 = d_1[MR]-a_1[M]([R]-[MR])$. The setting of parameters guarantees that $w_1 \approx w_2, w_3 \approx w_4$. Thus we define $r_w = (w_1+w_2)/(w_3+w_4)$. The dependence of net flux ratio r_w on $-\log\gamma_1$ shows a negative correlation with phase sensitivity.

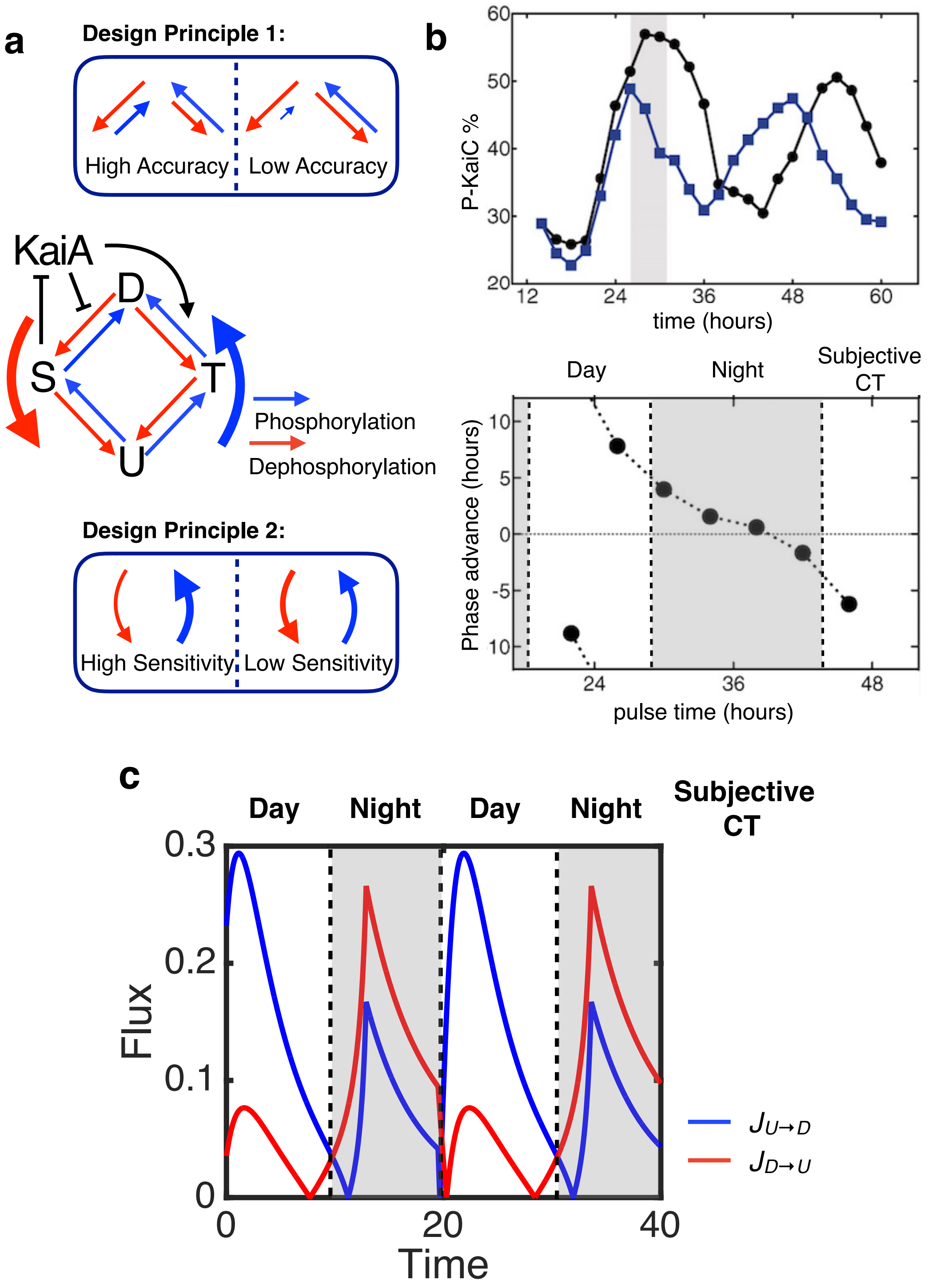

Supplementary Figure 10: Testing design principles with a KaiABC oscillator model. All kinetic rates are experimentally measured [1]. Results are then compared with experiments in [1]. **(a)** Schematic of the model together with the two design principles. Our theory propose that balancing the forward and backward transition leads to low phase diffusion while unevenly distributing net flux favors higher sensitivity. **(b)** Experimental results of *in vitro* Kai system. Adapted from [1]. Phosphorylation oscillation data (ATP%=100%) were used to estimate D in the system [2] and define subjective day and night (rise/drop in P-KaiC%). PRC against an ADP pulse shows larger phase shifts (higher sensitivity) during the subjective day. **(c)** The coarse-grained phosphorylation and dephosphorylation net flux $J^{(\text{net})}_{U\to D}$ (blue line) and $J^{(\text{net})}_{D\to U}$ (red line) are computed during the PdP cycle. Our design principle would predict phase sensitivity to be higher during subjective day where the backward-to-forward net flux ratio r_w is smaller. This is supported by the experimentally measured PRC (panel **b**) in [1].

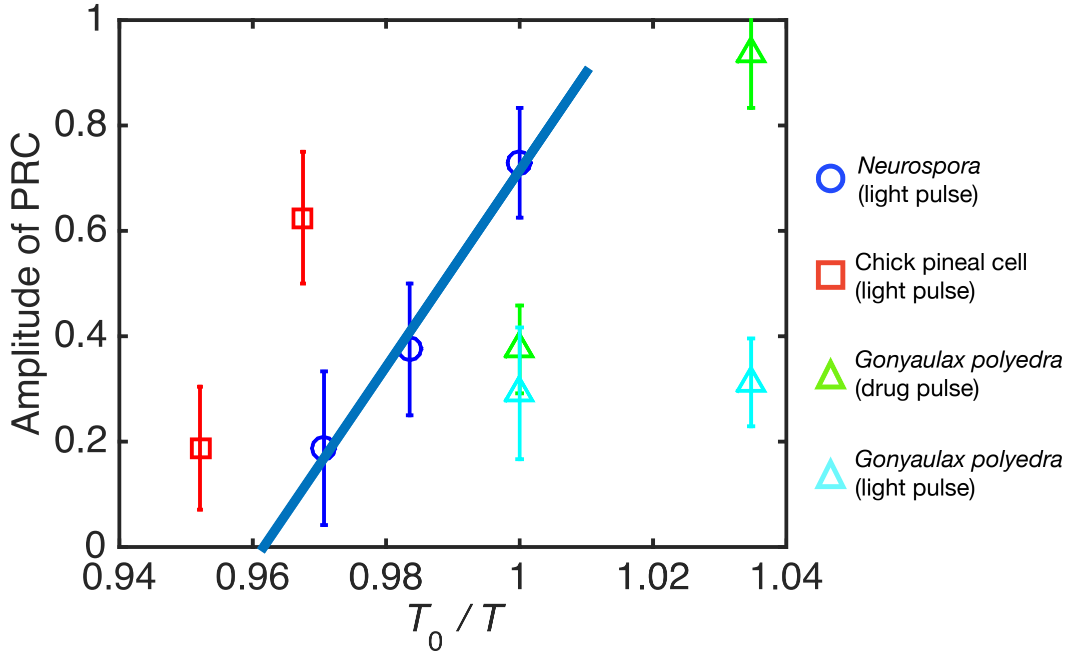

Supplementary Figure 11: Existing phase resetting experiments. PRC against light and drug pulse is measured at different ambient temperature in *Neurospora* (blue circle) [3], the unicellular dinoagellate *Gonyaulax polyedra* (green and cyan triangles) [4] and chick pineal cell (red square) [5]. Error bars represent standard deviation estimated from original experiments. Except for the case of light-PRC in *Gonyaulax polyedra* (cyan) where no significant temperature dependence was observed, lowering temperature (smaller γ or more irreversible nonequilibrium reaction loop) in the other three cases increases the PRC amplitude. The slope of the line, fitted to the case of *Neurospora*, is given by $\lambda \approx 18$. According to our theory, PRC amplitude $\Delta\phi$ could be related to ambient temperature T through $\Delta\phi/(\Delta r/r_\mathrm{s}) \approx \chi \sim K_W \Delta W \sim K_W C_W(-\ln\gamma) = K_W C_W(\Delta G^{(0)}/k_B T_0) \times T/T_0$, where K_W, C_W are the scale factors of approximate linear relationships, and $\Delta G^(0)/k_B T_0$ is the free energy difference in units of thermal energy at room temperature. The relative deviation of the limit cycle induced by perturbation $\Delta r/r$ is of order 10^{-1} to 1 based on observations of resetting response trajectories in the experiments. Taking $K_W \approx 10^{-2}$ and $C_W \approx 10^2$ from our simulations as well $\Delta r/r$ and λ from the experiments, our theory would estimate $\Delta G^{(0)}/k_B T_0$ to be roughly $10^1 \sim 10^2$, which seems to be biologically reasonable.

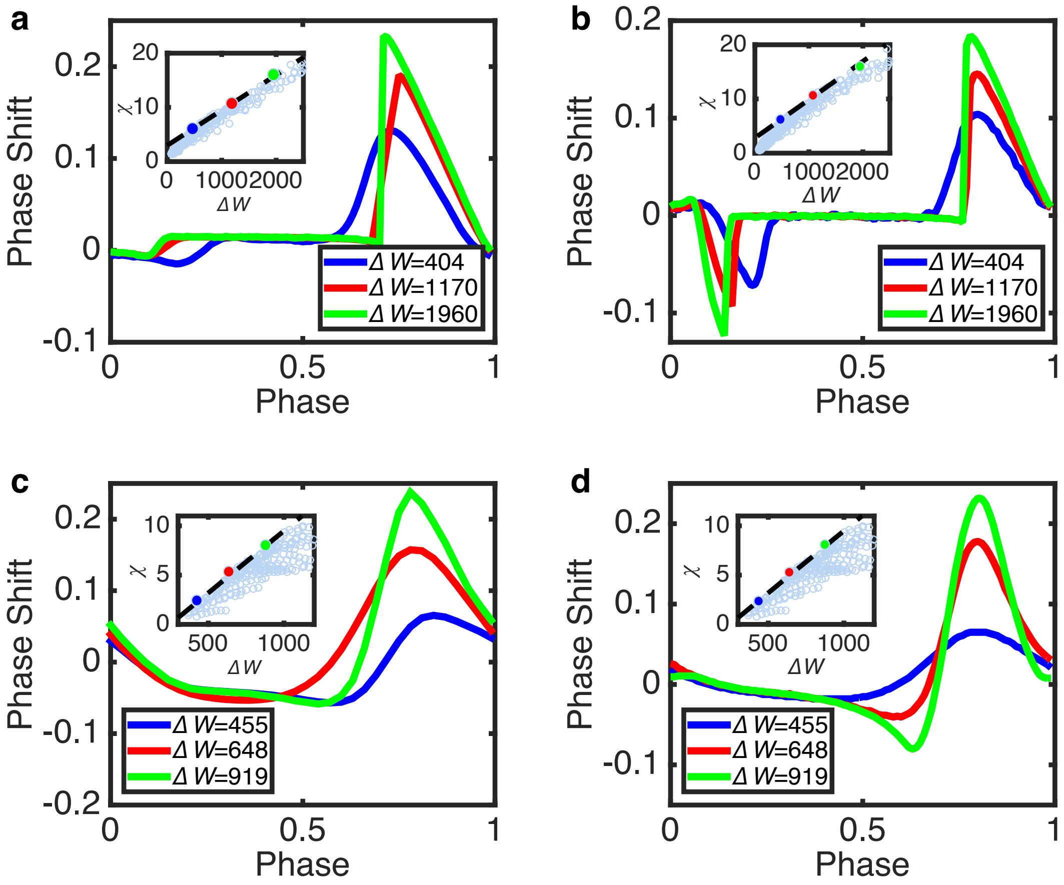

Supplementary Figure 12: Examples of parametric PRCs for different models and perturbations. In general, the amplitude of PRC increases with energy dissipation, as indicated by the enhanced phase sensitivity. This is a robust result independent of the model used and the specific parameter perturbed. **(a)** PRC of the Brusselator model against a 30% perturbation on k_1 for 0.05 period. **(b)** PRC of the Brusselator model against a 20% perturbation on k_3 for 0.05 period. The elongation of the dead zone is due to the nonuniform velocity along the limit cycle, i.e., the oscillator spends a large amount of time during the slow process. The parameters are: $\chi = 5.12, \gamma_1 = 0.19, \gamma_2 = 4.5 \times 10^{-3}$ $(blue)$; $\chi = 10.38, \gamma_1 = 0.12, \gamma_2 = 5.6 \times 10^{-3}$ (red); $\chi = 15.27, \gamma_1 = 0.11, \gamma_2 = 2.2 \times 10^{-3}$ $(green)$. **(c)** PRC of the AI model for a perturbation of a 75% drop in k_4 for 0.05 period. **(d)** PRC of the AI model for a perturbation doubling k_1 for 0.05 period. In the generic AI model, to which the Kai system belongs, PRCs are computed for different background levels of ATP/ADP ratio or equivalently different values of γ: $-\log\gamma = 3.2$ $(blue)$; 3.8 (red); 5.0 $(green)$. The free energy dissipation ΔW for each γ choice is given in the legend. Amplitude of PRC increases as sensitivity χ is enhanced by $-\log\gamma$ or ΔW as shown in the inset. The hollow circles in all the insets correspond to different choices of (γ_1, γ_2).

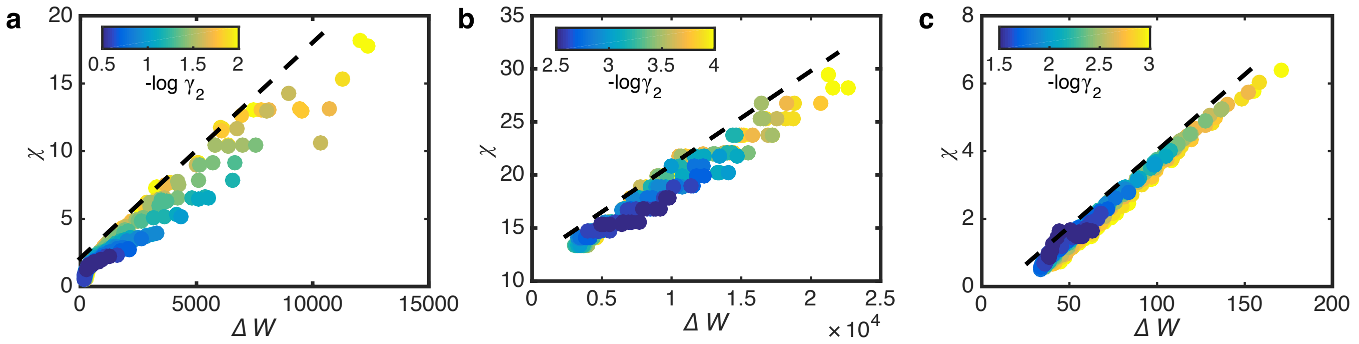

Supplementary Figure 13: The generality of results is confirmed in the Brusselator model. In particular, we fixed $k_1 = 1, k_{-3} = 1$ at equilibrium, and search for the remaining two degrees of freedoms $\lambda_1 = k_{-1}/k_1$ and $\lambda_2 = k_3/k_{-3}$. Different equilibrium reaction rates are chosen: (a) $\lambda_1 = 1, \lambda_2 = 10^{-1}$, (b)$\lambda_1 = 3, \lambda_2 = 10$, (c)$\lambda_1 = 10, \lambda_2 = 1$; but the linear enhancement of phase sensitivity by free energy cost is found in all the cases.

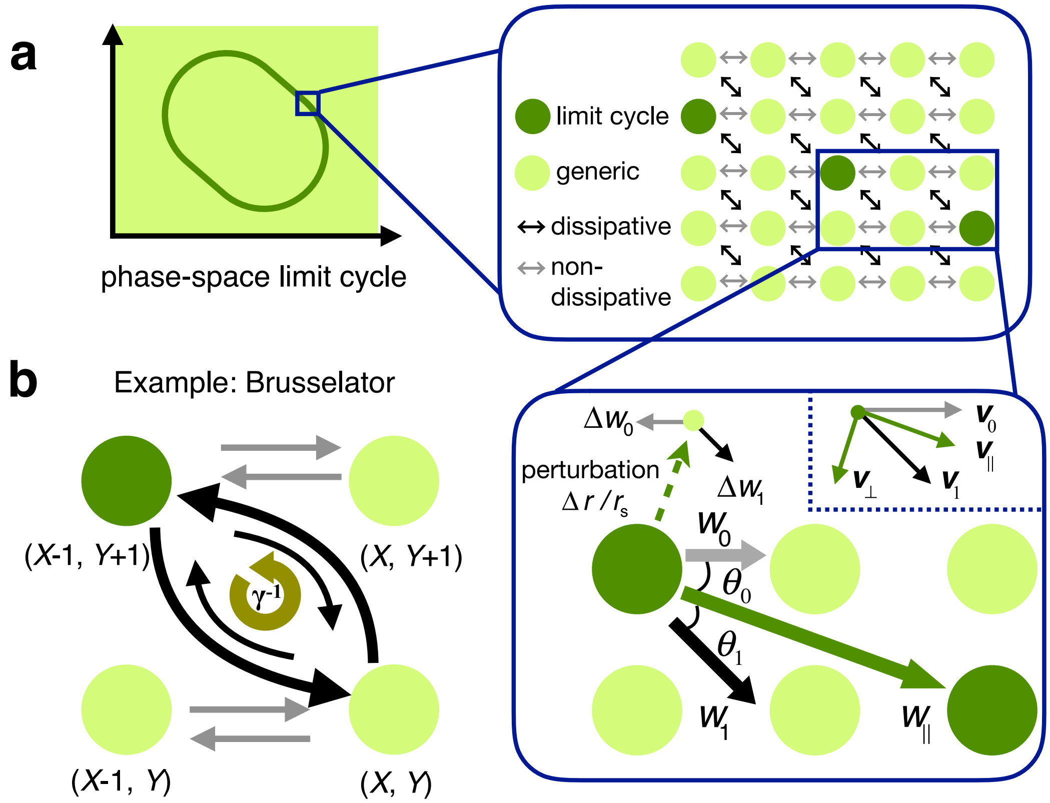

Supplementary Figure 14: Detailed illustration about the minimal model for limit cycle oscillation. (a) Microscopically, the progression along the limit cycle is due to the transition between phase-space states. In the minimal model, we confine the jumps to two possible directions $\boldsymbol{v}_0$ (non-dissipative reactions) and $\boldsymbol{v}_1$ (dissipative links). The direction of progression in the phase-space $\boldsymbol{v}_{\|}$ is determined by the transition flux w_0 and w_1 along both directions. **(b)** An example of the minimal model is the reversible Brusselator studied here. The non-dissipative links are achieved by reaction $A \leftrightarrows X$, and the dissipative links are realized by the nonequilibrium reaction loop. For this example, the angle between $\boldsymbol{v}_0$ and $\boldsymbol{v}_1$ is $\pi/4$.

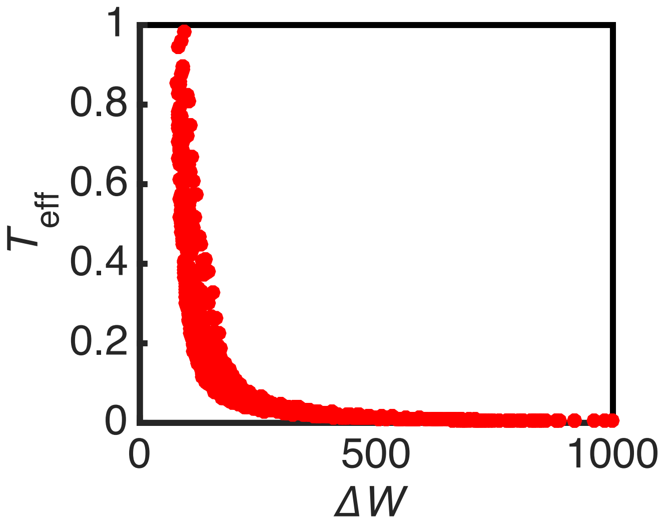

Supplementary Figure 15: Free energy dissipation lowers the effective temperature in Brusselator. By actively regulating T_{eff} of the system, energy cost makes it possible for biochemical oscillators to achieve both high accuracy and high sensitivity.

Supplementary Tables

Category	Process	Parameter	Value
Basal Rates (without KaiA)	T→U	k^0_{TU}	0.21 h^{-1}
	D→T	k^0_{DT}	0.00 h^{-1}
	D→S	k^0_{DS}	0.32 h^{-1}
	S→U	k^0_{SU}	0.11 h^{-1}
Maximal Effect of KaiA	U→T	k^A_{UT}	0.4790 h^{-1}
	T→D	k^A_{TD}	0.2130 h^{-1}
	S→D	k^A_{SD}	0.5057 h^{-1}
	U→S	k^A_{US}	0.0532 h^{-1}
	T→U	k^A_{TU}	0.0798 h^{-1}
	D→T	k^A_{DT}	0.1730 h^{-1}
	D→S	k^A_{DS}	-0.3200 h^{-1}
	S→U	k^A_{SU}	-0.1100 h^{-1}
Concentration Parameter	Concentration of KaiA causing half-maximal effect on KaiC	$K_{1/2}$	0.43 μM
	concentration of KaiA (total)	$[A]_{\text{total}}$	1.3 μM
	concentration of KaiC (total)	$[C]_{\text{total}}$	3.4 μM
Effect of ATP/ADP ratio	Effective relative affinity for ADP vs. ATP in kinase reactions	K_{rel}	1.0

Supplementary Table 1. Rate constants used in the simulation of Rust model. Phosphorylation and dephosphorylation processes are denoted by blue and red arrows respectively. We adopt the same parameter as in [1]. Apart from the introduction of the K_{rel} parameter to describe the relative effect of ADP on the kinase rates, all parameters use the values set previously by experimental data in [6]. This table is a copy of Table S1 in [1].

Supplementary Note 1. The Phase Reduction Method and Phase Response Curve for Biochemical Oscillator

We first introduce a powerful technique to study phase evolution of oscillation systems. Instead of dealing with the entire system, the phase reduction method, developed first by Kuramoto [7, 8], is used to reduce complex dynamic systems to a single phase variable. Here, we use the phase reduction method to study biochemical oscillators. Consider a mixture of N species $\{X_1, X_2, \ldots, X_N\}$ that interact, in some fixed volume V and at a constant temperature T, through M reaction channels. We specify the state of the system by a vector of concentration variables $\boldsymbol{x}(t) = (x_1(t), x_2(t), \ldots, x_N(t))$. The stochastic reaction kinetics of the system can be described by the chemical Langevin equation (CLE) [9] as follows:

$$\frac{\mathrm{d}x_i}{\mathrm{d}t} = F_i(\boldsymbol{x}, \mu) + f_i(\boldsymbol{x}, \mu, t) + \zeta_i(\boldsymbol{x}, \mu, t) \tag{1}$$

The reaction vector is $F_i(\boldsymbol{x}) = \sum_{j=1}^{M} v_{ji} a_j(\boldsymbol{x})$ where v_{ji} are the stoichiometric change of species i in the jth reaction, and $a_j\mathrm{d}t$ are the probability that j^{th} reaction will occur (once) during $t \to t + dt$. The term f_i represents an external perturbation, and the noise ζ_i can be expressed as:

$$\zeta_i(t) = (V)^{-1/2} \sum_{j=1}^{M} v_{ji} \sqrt{a_j(\boldsymbol{x})} \xi_j(t), \tag{2}$$

where M independent Gaussian white noise $\xi_j(t)$ are associated with M reaction channels, with $\langle \xi_j(t) \rangle = 0$ and $\langle \xi_j(t) \xi_k(t') \rangle = \delta_{jk} \delta(t - t')$.

In the absence of perturbation and internal noise, Supplementary Equation (1) are reduced to deterministic kinetic equations. Here, we consider the cases where the deterministic equations exhibit limit cycle behavior. For each state $\boldsymbol{x}^{\mathcal{L}}$ along the limit cycle $\mathcal{L}$ we can define a phase variable ϕ. For simplicity, $\phi(\boldsymbol{x}^{\mathcal{L}})$ is chosen to progress along $\mathcal{L}$ with a constant speed $\Omega = 2\pi/\tau$, where τ is the period. Following this definition, the deterministic phase evolution equation of (1) can be expressed as

$$\frac{\mathrm{d}\phi}{\mathrm{d}t} \equiv \Omega = \nabla_{\boldsymbol{x}} \phi \cdot F(\boldsymbol{x}, \mu). \tag{3}$$

Now we consider the phase shift incurred by a parametric perturbation $\mu \to \mu + \Delta\mu$ for duration Δt in our model. In the linear response regime, the perturbation can be written as $f(t) = \partial_\mu F \Delta\mu$ $(0 \le t \le \Delta t)$, and the perturbed phase evolution equation becomes

$$\frac{d\phi}{dt} = (\nabla_{\boldsymbol{x}} \phi)_\mu \cdot \left(F(\boldsymbol{x}, \mu) + \frac{\partial F(\boldsymbol{x}, \mu)}{\partial \mu} \Delta\mu \right) \tag{4}$$

$$= \Omega + Z(\phi)_\mu \Delta\mu \tag{5}$$

where

$$Z(\phi)_\mu = (\nabla_{\boldsymbol{x}} \phi)_\mu \cdot \frac{\partial F(\boldsymbol{x}(\phi), \mu)}{\partial \mu}. \tag{6}$$

The subscript μ indicates that all the quantities are measured under parameter μ. It should be noted that $Z(\phi)$, known as the infinitesimal PRC function, is exactly the PRC normalized by perturbation intensity $\Delta\mu$ and duration Δt when they are sufficiently small [10].

Different external stimuli can have different PRC shapes since the second term in the right hand side of Supplementary Equation (6) depends on the specific perturbations. But the first term $\nabla_{\boldsymbol{x}} \phi$, characterizing the topological structure of the isochrons (see main text Fig. 1), is an intrinsic property of the limit cycle oscillator. Clearly, larger $\nabla_{\boldsymbol{x}} \phi$ would produce larger phase shifts for the same deviation from the limit cycle.

Supplementary Note 2. Phase Sensitivity and Entrainability

Consider a biochemical oscillator exposed to external periodic signal $\epsilon p(\omega, t)$ (e.g., temperature, light, etc.) with period $2\pi/\omega$ and intensity ϵ. We further assume that the stimulus is applied on parameter μ. Interpreting this

periodic driving force as time-dependent perturbation $f(t) = \epsilon\partial_\mu F p(\omega, t)$ in Supplementary Equation Supplementary Equation (1), we obtain the following dynamic equation for the phase ϕ

$$\frac{d\phi}{dt} = \Omega + \epsilon Z(\phi)_\mu \cdot p(\omega, t). \tag{7}$$

In the main text, we studied the entrainment of the internal oscillator towards the external periodic signal when $\omega = \Omega$. In general, there might be mismatch between the frequency of the oscillator (Ω) and that of the external driving signal (ω). What is the range of synchronizable frequencies for a certain oscillator? By introducing phase difference $\psi = \phi - \omega t$, and averaging over a period (since ψ is a slow variable for small ϵ), we have

$$\frac{d\psi}{dt} = (\Omega - \omega) + \epsilon E(\psi) \tag{8}$$

where $E(\psi) = \frac{1}{2\pi}\int_0^{2\pi} Z(\psi + \theta)p(\theta)d\theta$. Entrainment to external periodicity occurs when $\dot{\psi} = 0$ has one stable fixed point. This condition places constraints on the signal frequency

$$\epsilon E(\psi)_{\min} + \Omega < \omega < \epsilon E(\psi)_{\max} + \Omega \tag{9}$$

Accordingly, we may define entrainability of a biochemical oscillator as $\mathcal{E} = E(\psi)_{\max} - E(\psi)_{\min}$. How is $\mathcal{E}$ related to phase sensitivity χ? We suggest that high phase sensitivity increases the amplitude of PRC, therefore enhancing entrainability $\mathcal{E}$. For example, if the signals take the form of square wave, i.e. the 2π periodic function $p(\theta)$ is a Heaviside step function between $-\pi$ and π, then $E(\psi) = \frac{1}{2\pi}\int_0^{\pi} Z(\psi + \theta)d\theta$, which integrates the area under PRC. One can imagine that as long as the shape of PRC remains approximately the same, $\mathcal{E} = E(\psi)_{\max} - E(\psi)_{\min}$ should scale with the PRC amplitude.

Supplementary Note 3. Diffusive Phase Dynamics

Here, we consider the biochemical oscillator in the presence of internal noise. Interpreting the random "forces" $\zeta_i(t)$ in Supplementary Equation 1 as a time-dependent perturbation, we can immediately write down the stochastic phase evolution equation (first-order)

$$\frac{\mathrm{d}\phi}{\mathrm{d}t} = \Omega + \nabla_{\boldsymbol{x}}\phi \cdot \boldsymbol{\zeta}(\boldsymbol{x}^{\mathcal{L}}(\phi), t) \equiv \Omega + g(\phi, t) \tag{10}$$

Here, $g(\phi, t)$ is a τ-periodic function of ϕ but stochastically dependent on t, with $\langle g(\phi, t)g(\phi, t')\rangle \equiv D(\phi)\delta(t - t')$.

It is straightforward to see that a Langevin equation of the form (10) is equivalent to a Fokker-Planck equation (in Stratonovich sense)

$$\frac{\partial P(\phi, t)}{\partial t} = -\frac{\partial}{\partial\phi}\Big(\Omega + \frac{1}{2}D'(\phi)\Big)P + \frac{\partial^2}{\partial\phi^2}(DP) \tag{11}$$

We then introduce a new variable $\delta\phi = \phi - \Omega t$ to describe phase fluctuation. Let $\widetilde{P}(\delta\phi, t)$ denote the probability distribution for $\delta\phi$, then it immediately follows that

$$\frac{\partial\widetilde{P}}{\partial t} = -\frac{1}{2}\frac{\partial D(\Omega t + \delta\phi)}{\partial(\delta\phi)}\widetilde{P} + \frac{\partial^2}{\partial(\delta\phi)^2}(D(\Omega t + \delta\phi)\widetilde{P}) \tag{12}$$

In the weak noise limit, $\widetilde{P}$ is a slowly varying function of t while $\partial D/\partial(\delta\phi)$ and D fluctuate much faster so that they may safely be time averaged over the period τ. Since the average of $\partial D/\partial(\delta\phi)$is zero, we are left with a simple diffusion equation,

$$\frac{\partial\widetilde{P}}{\partial t} = D_\phi\frac{\partial^2\widetilde{P}}{\partial(\delta\phi)^2} \tag{13}$$

where $D_\phi = (2\pi)^{-1}\int_0^{2\pi} D(\phi')d\phi'$.

Supplementary Note 4. Phase Diffusion and Coherence Time

Given the Langevin dynamics of phase ϕ (Supplementary Equation 10), we next calculate for a sinusoidal wave, $x = e^{i\phi} + \text{c.c.}$, the correlation function $C(t) = \langle x(t+s)x(s)\rangle_s$ in which the average takes the form $\langle ...\rangle_s = \lim\limits_{S\to\infty} \frac{1}{S}\int_0^S ...ds$. Notice that any oscillatory part with respect to s vanishes in the long time average. Thus, the autocorrelation function is simplified to

$$\langle x(t+s)x(s)\rangle_s = \Big\langle \Big[\exp(i\Omega(t+s) + i\int_0^{t+s} g(t')dt') + \text{c.c.}\Big]\Big[\exp(i\Omega s + i\int_0^{s} g(t')dt') + \text{c.c.}\Big]\Big\rangle_s \tag{14}$$

$$= e^{i\Omega t}\Big\langle \exp(i\int_0^{t} g(t')dt')\Big\rangle + \text{c.c.} \tag{15}$$

In the last equation, we take off the subscript s since averaging over s is equivalent to the ensemble average. The exponent $\delta\phi(t) = \int_0^t g(t')dt'$ can be interpreted as the phase fluctuation around the mean phase Ωt after time t. It is clear, from the phase diffusion dynamics (Supplementary Equation 13), that the probability distribution of $\delta\phi$ evolves as

$$P(\delta\phi, t) = \frac{1}{\sqrt{4\pi D_\phi t}} \exp\Big[-\frac{(\delta\phi)^2}{4D_\phi t}\Big], \tag{16}$$

if we assume D_ϕ to be a constant for simplicity. After some straightforward calculations, the expression for autocorrelation can be divided into two parts. The ensemble average

$$\Big\langle \exp(i\int_0^{t} g(t')dt')\Big\rangle = \int \exp(i\delta\phi)P(\delta\phi)d(\delta\phi) = \exp(-D_\phi t) \equiv \exp(-t/\tau_c) \tag{17}$$

gives the exponential decay of the autocorrelation function while $e^{i\Omega t}$ gives its oscillatory part. Thus, we show that the autocorrelation function of any observable x is indeed in the form of main text Eq. (2). In general, we expect that the coherence time would take the form $\tau_c = cD_\phi^{-1}$, where c is a constant depending on the specific waveform of the oscillation ($c = 1$ for sinusoidal wave).

Supplementary Note 5. Details of the Models

A. Activator-Inhibitor Model

The main components of the model are the activator R and the inhibitor X (Supplementary Figure 1, see also [2] for details). The kinetics can be described by:

$$\begin{aligned}
\frac{d[R]}{dt} &= k_0[M_p] + k_1 S - k_2[X][R] \\
\frac{d[X]}{dt} &= k_3[M_p] - k_4[X] \\
\frac{d[M]}{dt} &= f_2[M_pK] + d_1[MR] - a_1[M][R] - f_{-2}[M][K] \\
\frac{d[MR]}{dt} &= a_1[M][R] + f_{-1}[M_p][R] - (f_1 + d_1)[MR] \\
\frac{d[M_p]}{dt} &= f_1[MR] + d_2[M_pK] - f_{-1}[M_p][R] - a_2[M_p][K] \\
\frac{d[M_pK]}{dt} &= a_2[M_p][K] + f_{-2}[M][K] - (f_2 + d_2)[M_pK]
\end{aligned} \tag{18}$$

with mass conservation constraints: $[M] + [MR] + [M_p] + [M_pK] = M_T$, $[K] + [M_pK] = K_T$, where M_T and K_T denote the total concentrations of enzyme M and phosphatase K. We take symmetric parameters: $k_0 = k_1 = k_3 = 1$, $k_4 = 0.5$, $S = 0.4$, $K_T = 1$, $M_T = 10$, $a_2 = 100$, $f_1 = f_2 = d_1 = d_2 = 15$. The PdP regulated rates are $a_1 = a_{1,0}[\text{ATP}], f_{-1} = f_{-1,0}[\text{ADP}]$ and $f_{-2} = f_{-2,0}[\text{P}_i]$ (see caption of Supplementary Figure 1 for details). The irreversibility parameter $\gamma = (d_1 d_2 f_{-1} f_{-2})/(a_1 a_2 f_1 f_2) = \gamma_1\gamma_2$.

Note that the reverse reactions in the AI motif are very weak so that they can be neglected in studying the kinetics and the free energy dissipation in the PdP cycle. However, for thermodynamic consistency, we can include all the reverse reactions outside the PdP cycle and compute the additional dissipation on these reactions outside of the PdP cycle.

There are different ways to introduce the reverse reactions outside of the PdP cycle. One is proposed in Appendix F of Barato and Seifert[11], who directly write down the reverse reactions of those irreversible processes, and assign them with some small rates ϵ. Another approach is to introduce a pool of "inactive" activators and inhibitors R_i and X_i. The generation/degradation of R and X in the original model can be regarded as transformation from/to R_i and X_i following the Michaellis-Menten (MM) kinetics. The complete activator-inhibitor model can therefore be described by a set of chemical reactions as follows

$$\begin{aligned}
\text{PdP cycle:}\quad & M + R + \text{ATP} \underset{d_1}{\overset{a_{1,0}}{\rightleftharpoons}} M\cdot R\cdot \text{ATP} \underset{f_{-1,0}}{\overset{f_1}{\rightleftharpoons}} M_p + R + \text{ADP}, \\
& M_p + K \underset{d_2}{\overset{a_2}{\rightleftharpoons}} M_p\cdot K \underset{f_{-2,0}}{\overset{f_1}{\rightleftharpoons}} M + K + P_i
\end{aligned} \tag{19}$$

$$\begin{aligned}
\text{AI motif (direct reverse):}\quad & \varnothing \underset{k_{-1}}{\overset{k_1 S}{\rightleftharpoons}} R, \quad X \underset{\epsilon_4}{\overset{k_4}{\rightleftharpoons}} \varnothing, \quad X + R \underset{k_{-2}}{\overset{k_2}{\rightleftharpoons}} X \\
& M_p \underset{k_{-0}}{\overset{k_0}{\rightleftharpoons}} R, \quad M_p \underset{k_{-3}}{\overset{k_3}{\rightleftharpoons}} X
\end{aligned} \tag{20}$$

$$\begin{aligned}
\text{AI motif (MM):}\quad & R_i \underset{k_{-1}}{\overset{k_1}{\rightleftharpoons}} R, \quad X \underset{k_{-4}}{\overset{k_4}{\rightleftharpoons}} X_i, \quad X + R \underset{k_{2d}}{\overset{k_{2a}}{\rightleftharpoons}} X\cdot R \underset{k_{-2}}{\overset{k_2}{\rightleftharpoons}} X + R_i, \\
& M_p + R_i \underset{k_{0d}}{\overset{k_{0a}}{\rightleftharpoons}} M_p\cdot R_i \underset{k_{-0}}{\overset{k_0}{\rightleftharpoons}} M_p + R, \quad M_p + X_i \underset{k_{3d}}{\overset{k_{3a}}{\rightleftharpoons}} M_p\cdot X_i \underset{k_{-3}}{\overset{k_3}{\rightleftharpoons}} M_p + X.
\end{aligned} \tag{21}$$

It is straightforward to write down the deterministic kinetic equations for these elementary reactions. The corresponding chemical master equation can also be written down in a similar manner as in [11], even with the negligible reverse reactions.

In our simulations, we chose parameter values $k_{-i}/k_i = \epsilon_4 = 10^{-4} (i = 0, 1, \ldots, 3)$ for the direct reverse scheme and $[X_i] = [R_i] = 1, k_{-1} = 10^{-2}k_1 = 4\times 10^{-3}, k_{-4} = 10^{-2}k_4 = 5\times 10^{-3}, k_{-2} = 10^{-6}k_2 = 0.1, k_{2a} = 10^{-4}k_{2d} = 10^6, k_0 = k_3 = 3, k_{-0} = k_{-3} = 3\times 10^{-4}, k_{0a} = k_{0d} = k_{3a} = k_{3d} = 10^4$ for the Michaelis-Menten scheme. Since the reverse rates are negligible and the MM rates are chosen to separate the timescale, they don't have any significant effect on the original dynamics of the system, and therefore the phase sensitivity and diffusion. On the other hand, the total energy dissipation of the system depends on the value of reverse reaction rates. However, for reasonable choices of the reverse reaction rates as used here, the energy dissipation due to these processes outside of the PdP cycle remains relatively small, as shown in Supplementary Figure 2. More importantly, the energy cost outside of the PdP cycle does not vary much with γ as shown in Supplementary Figure 2 for both the direct reverse scheme and the MM scheme. In this paper, we are primarily interested in the part of dissipation that affects the oscillatory behavior of the system when ATP and/or ADP concentrations are varied in the PdP cycle (Supplementary Figure 2). Therefore, the simplified version of AI model (irreversible AI motif) is a reasonable approximation to study the relationship between performance of the oscillation and the free energy cost within the PdP cycle.

B. Brusselator

The reversible Brusselator is described in the main text. The deterministic kinetic equations for reactants X and Y are:

$$\begin{aligned}
\frac{d[X]}{dt} &= k_1[A] - k_{-1}[X] - k_2^0[B][X] + k_{-2}^0[D][Y] + k_3[X]^2[Y] - k_{-3}[X]^3 \\
\frac{dY}{dt} &= k_2^0[B][X] - k_{-2}^0[D][Y] - k_3[X]^2[Y] + k_{-3}[X]^3
\end{aligned} \tag{22}$$

In this model, concentration $[B]$ and $[D]$ are fixed and absorbed in the reaction rate constant, resulting in pseudo first order rates with $k_2 = k_2^0[B]$ and $k_{-2} = k_{-2}^0[D]$. We choose parameter values $[A] = 1, k_1 = k_{-1} = 1$ and set $k_2 = k_3 = k_{-2} = k_{-3} = 1$ at equilibrium in the simulation to highlight the effects of irreversibility γ_1 and γ_2 introduced to the system.

To test the generality of our main text Eq. (6), we also studied different implementations of γ_1, γ_2 (Supplementary Figure 4) and various kinetic rates (Supplementary Figure 13) in the Brusselator model. We confirmed the linear enhancement of phase sensitivity by energy dissipation is not just a special case for certain kinetic rates or γ_1, γ_2

partitions.

C. Simulation Methods

The Gillespie algorithm[12] is used for both models to simulate the stochastic reaction kinetics. We use volume $V = 100$ for all calculations. For given kinetic rates and the volume V, we simulated $N = 300$ trajectories starting from the same initial condition over 25 periods. We compute autocorrelation function for each trajectory and then average over N trajectories to get $C_{xx}(t)$ for further analysis. In the absence of external driving, we fit $C_{xx}(t)$ with main text Eq. (2), i.e. a damped oscillation, and calculate τ_c/τ as a proxy for the dimensionless diffusion constant D. For the periodically driven oscillators, we infer D from the steady state phase variance described in the main text Result Section C.

Supplementary Note 6. Stochastic Analysis of Limit Cycle Oscillation

Here, we study the stochastic limit cycle oscillation analytically. For simplicity, we start with a 2-dimensional system, such as the reversible Brusselator described above. In the absence of noise, the equations governing the evolution of the system are given by the deterministic part of CLE (Supplementary Equation 1) in Supplementary Note 1.

For any generic two-dimensional system undergoing a Hopf bifurcation, it is known that there exist an invertible coordinate change $\mathcal{T} : (x_1, x_2) \to (r, \theta)$ and parameter change locally (near the fixed point and the bifurcation point) transforming its kinetic equations $dx_i/dt = F_i(x_1, x_2; \alpha)$ into the Stuart-Landau normal form [13]:

$$\begin{aligned} \frac{\mathrm{d}r}{\mathrm{d}t} &= \mu r - \beta_1 r^3 \\ \frac{\mathrm{d}\theta}{\mathrm{d}t} &= \omega + \beta_2 r^2 \end{aligned} \tag{23}$$

Applying the same transformation $\mathcal{T}$ to the CLE, we then obtain the stochastic Stuart-Landau equation (SLE) with the original normal form plus the transformed noise terms expressed in the polar coordinate:

$$\begin{aligned} \frac{\mathrm{d}r}{\mathrm{d}t} &= \mu r - \beta_1 r^3 + \frac{1}{\sqrt{V}} \sum_{j=1}^{M} q_{rj}\ \xi_j(t) \\ \frac{\mathrm{d}\theta}{\mathrm{d}t} &= \omega + \beta_2 r^2 + \frac{1}{\sqrt{V}} \sum_{j=1}^{M} q_{\theta j}\ \xi_j(t) \end{aligned} \tag{24}$$

where $q_{rj} = (\tilde{v}_{j1} \cos\theta + \tilde{v}_{j2} \sin\theta)\sqrt{a_j}$ and $q_{\theta j} = (-\tilde{v}_{j1} \sin\theta + \tilde{v}_{j2} \cos\theta)\sqrt{a_j}/r$, and $\tilde{v}_{ji}$ are transformed by $\mathcal{T}$ from v_{ji} (stoichiometric change of species i in the j reaction, see Section Supplementary Note 1). Here $\mu, \omega, \beta_1, \beta_2$ are parameters associated with reaction rate constants, i.e., original parameters in CLE. Unlike the deterministic version of SLE, here r and θ are coupled to each other through internal noise, making it almost impossible for direct analysis.

However, we can use stochastic averaging method [14] to derive the approximate equations

$$\begin{aligned} \frac{\mathrm{d}r}{\mathrm{d}t} &= \mu r - \beta_1 r^3 + \frac{q_r}{\sqrt{V}} \xi_r(t), \\ \frac{\mathrm{d}\theta}{\mathrm{d}t} &= \omega + \beta_2 r^2 + \frac{q_\theta}{\sqrt{V} r} \xi_\theta(t), \end{aligned} \tag{25}$$

where $q_r^2 = 1/2\pi \int_0^{2\pi} \mathrm{d}\theta \sum_j q_{rj}^2$ and $q_\theta^2/r^2 = 1/2\pi \int_0^{2\pi} \mathrm{d}\theta \sum_j q_{\theta j}^2$ are the average noise over a period. Equivalently, ξ_r and ξ_θ are two independent, "unit normal" random variable. For small oscillation amplitudes, the leading order in q_r^2 and q_θ^2 are both $Q = \sum_j (\tilde{v}_{j1}^2 + \tilde{v}_{j2}^2) a_j^{(0)}/2$ where the superscript 0 indicates the values are measured at the fixed point. The analysis so far are discussed in a 2D system; however, the results can be well extended to any higher dimensional system whose central manifold is also 2 dimensional [13].

For the rest of this section, we explicitly demonstrate how the stochastic SLE is derived from the original CLE

using the reversible Brusselator as an example. The chemical Langevin equation for the Brusselator model reads

$$\begin{aligned}\frac{dx}{d\hat{t}} &= a - (1+b_+)x + b_-y + c_+x^2y - c_-x^3 + \zeta_x(\hat{t}) \\ \frac{dy}{d\hat{t}} &= b_+x - b_-y - c_+x^2y + c_-x^3 + \zeta_y(\hat{t})\end{aligned} \tag{26}$$

where $\hat{t} = k_{-1}t$ is the dimensionless time. Other coefficients are given by: $a = k_1[A]/k_{-1}$, $b_+ = k_2/k_{-1}$, $b_- = k_{-2}/k_{-1}$, $c_+ = k_3/k_{-1}$, $c_- = k_{-3}/k_{-1}$. Here, reaction rates k_i are the same as defined in deterministic reaction equations, and $[A]$ is the concentration of A. The internal noise ζ_x and ζ_y, arising from six chemical reaction channels, are

$$\begin{aligned}\zeta_x &= (V)^{-1/2}\Big[\sqrt{a}\xi_1(\hat{t}) - \sqrt{x}\xi_2(\hat{t}) - \sqrt{b_+x}\xi_3(\hat{t}) + \sqrt{b_-y}\xi_4(\hat{t}) + \sqrt{c_+x^2y}\xi_5(\hat{t}) - \sqrt{c_-x^3}\xi_6(\hat{t})\Big] \\ \zeta_y &= (V)^{-1/2}\Big[\sqrt{b_+x}\xi_3(\hat{t}) - \sqrt{b_-y}\xi_4(\hat{t}) - \sqrt{c_+x^2y}\xi_5(\hat{t}) + \sqrt{c_-x^3}\xi_6(\hat{t})\Big]\end{aligned} \tag{27}$$

where $\xi_i(\hat{t})$ are temporally uncorrelated, statistically independent, unit variance Gaussian white noise.

The fixed point is $x_0 = a$, $y_0 = a(b_+ + c_-a^2)/(a^2c_+ + b_-)$. We choose $\alpha = b_+ - b_c$ to be the control parameter, and express (26) in terms of the deviation $(u, v) = (x - x_0, y - y_0)$ as

$$\frac{d}{d\hat{t}}\begin{pmatrix}u\\v\end{pmatrix} = A(\alpha)\begin{pmatrix}u\\v\end{pmatrix} + G(u, v; \alpha) + \zeta(x_0, y_0, u, v; \alpha) \tag{28}$$

where $A(\alpha)$ is the Jacobian matrix, and $G(u, v; \alpha)$ contains Taylor expansions in u, v starting with at least quadratic terms.

Next, we perform a transformation to reduce the deterministic part to the normal form of a Hopf bifurcation[13], and simultaneously transform the noise terms. This leads to equation

$$\frac{dz}{d\hat{t}} = (\mu + i\omega)z - (\beta_1 - i\beta_2)|z|^2z + \eta_1(\hat{t}) + i\eta_2(\hat{t}) \tag{29}$$

where $z = z_1 + iz_2 = re^{i\theta}$ whose real and complex parts are the transformed variable from u, v.

The entire process is tedious (see [13] for details), so we only list the results here:

$$\begin{aligned}\mu(\alpha) &= \frac{1}{2}\cdot\frac{a^2c_+ - b_-}{a^2c_+ + b_-}\cdot\alpha \\ \omega(\alpha) &= \sqrt{A^2 - \mu^2} \\ \beta(\alpha) = \beta_1(\alpha) - i\beta_2(\alpha) &= \frac{g_{20}g_{11}(2\lambda + \bar{\lambda})}{2|\lambda|^2} + \frac{|g_{11}|^2}{\lambda} + \frac{|g_{02}|^2}{2(2\lambda - \bar{\lambda})} + \frac{g_{21}}{2}\end{aligned} \tag{30}$$

where $\lambda = \mu(\alpha) + i\omega(\alpha)$ is the eigenvalue of $A(\alpha)$ and others are given by

$$\begin{aligned}A^2 &= a^2c_+ + b_- \\ g_{20} &= \frac{-i\lambda}{\omega}\Big[c_+y_0(\alpha) - 3ac_- - 2ac_+(1 + \frac{\bar{\lambda}}{A^2})\Big] \\ g_{11} &= \frac{-i\lambda}{\omega}\Big[c_+y_0(\alpha) - 3ac_- - 2ac_+(1 + \frac{\mu}{A^2})\Big] \\ g_{02} &= \frac{-i\lambda}{\omega}\Big[c_+y_0(\alpha) - 3ac_- - 2ac_+(1 + \frac{\lambda}{A^2})\Big] \\ g_{21} &= \frac{-i\lambda}{\omega}\Big[-3(c_- + c_+) - c_+\frac{\lambda + 2\bar{\lambda}}{A^2}\Big]\end{aligned} \tag{31}$$

The leading terms of η_1 and η_2 in (29) give

$$\eta_1 = \frac{1}{2\sqrt{V}}\Big(\sqrt{a}\xi_1(\hat{t}) - \sqrt{x_0}\xi_2(\hat{t}) - \sqrt{b_+x_0}\xi_3(\hat{t}) + \sqrt{b_-y_0}\xi_4(\hat{t}) + \sqrt{c_+x_0^2y_0}\xi_5(\hat{t}) - \sqrt{c_-x_0^3}\xi_6(\hat{t})\Big)$$
$$\eta_2 = \frac{A}{2\sqrt{V}}\Big(\sqrt{a}\xi_1(\hat{t}) - \sqrt{x_0}\xi_2(\hat{t})\Big) \tag{32}$$

with $\langle\eta_i(\hat{t})\eta_j(\hat{t}')\rangle = Q_{ij}V^{-1}\delta(\hat{t}-\hat{t}')$.

Supplementary Note 7. Isochron of Stuart-Landau Equation

To study the relation between entropy production rate and phase sensitivity in a stochastic SLE described above, we first use the mean-field SLE Equation (23) to analyze the isochron structure of its limit cycle.

For $\mu > 0$, the system starts to oscillate with a mean amplitude $r_\mathrm{s} = \sqrt{\mu/\beta_1}$ and angular velocity $\Omega = \omega + \beta_2 r_\mathrm{s}^2$. The phase ϕ is chosen to agree with θ on the limit cycle, i.e. $\phi(r_\mathrm{s}, \theta) = \theta$. Next, we derive the expression of isochron, which is a set of points with the same ϕ.

Consider an orbit start at (r_0, θ_0) outside the limit-cycle. The radial evolution is autonomous, following

$$r(t)^2 = \frac{r_\mathrm{s}^2}{1+\kappa e^{-2\mu t}}, \tag{33}$$

where κ is a constant determined by initial condition $r(0) = r_0$, and $(r_0/r_\mathrm{s})^2 = 1/(1+\kappa)$.

Suppose the point eventually approaches the limit cycle, and converges with the point of initial phase value θ. This leads to the result

$$\theta = \theta_0 + \int(\dot{\theta} - \omega_s)\mathrm{d}t = \theta_0 + \int_0^\infty \beta_2(r(t)^2 - r_\mathrm{s}^2)\mathrm{d}t$$
$$= \theta_0 - \frac{\beta_2}{\beta_1}\ln(\frac{r_0}{r_\mathrm{s}}) \tag{34}$$

Recall that the definition of phase is extended to the entire attracting basin of a limit-cycle oscillator by the asymptotic phase convention. The isochorn can thus be calculated by setting $\theta = \text{const.}$, which leads to main text Eq. (12)

$$\phi(r,\theta) = \theta - \frac{\beta_2}{\beta_1}(\ln r - \frac{1}{2}\ln\frac{\mu}{\beta_1}) \tag{35}$$

Supplementary Note 8. Energy Dissipation Enhances Phase Sensitivity

Now we turn to the stochastic SLE (25) to compute the entropy production (minimal free energy dissipation) of the system. Let $\Delta = Q/V$, the Fokker-Plank equation for (25) is given by [2]

$$\frac{\partial P}{\partial t} = -\frac{1}{r}\frac{\partial}{\partial r}\Big(rJ_r\Big) - \frac{1}{r}\frac{\partial}{\partial\theta}J_\theta$$
$$= -\frac{1}{r}\frac{\partial}{\partial r}\Big(r(\mu r - \beta_1 r^3)P - r\Delta\partial_r P\Big) - \frac{1}{r}\frac{\partial}{\partial\theta}\Big(r(\omega+\beta_2 r^2)P - \Delta r^{-1}\partial_\theta P\Big) \tag{36}$$

where $\boldsymbol{J} = \boldsymbol{F}P - \mathbb{D}\nabla P$ is the probability flux; $\boldsymbol{F}$ and $\mathbb{D}$ are the force vector and noise matrix of (25) respectively. The steady state probability distribution is

$$P^{\mathrm{ss}}(r,\theta) = A\exp\Big[-\frac{(\beta_1 r^4/4 - \mu r^2/2)}{\Delta}\Big] \tag{37}$$

The normalization coefficient is given by $A^{-1} = (2\pi)\int\exp[-(\beta_1 r^4/4 - \mu r^2/2)/\Delta]r\mathrm{d}r$. The definition of phase requires that the amplitude fluctuation is much smaller than $r_\mathrm{s}^2 = \mu/\beta_1$, leading to the first constraint $\rho = \mu/\sqrt{2\beta_1\Delta} \gg 1$ [2].

The entropy production rate can be computed from FPE as

$$\dot{S}_{\text{tot}} = \int \frac{\boldsymbol{J}^T \mathbb{D}^{-1} \boldsymbol{J}}{P} \mathrm{d}\boldsymbol{x} = \iint \frac{\|\boldsymbol{J}\|^2}{\Delta P} r \mathrm{d}r \mathrm{d}\theta \tag{38}$$

In this case, $J_r = r(\mu r - \beta_1 r^3)P^{\text{ss}} - r\Delta\partial_r P^{\text{ss}} = 0$, $J_\theta = r(\omega + \beta_2 r^2)P^{\text{ss}}$. Thus, the minimal dissipation rate is given by ($k_B T$=1)

$$\dot{W} = \iint \frac{r^2(\omega + \beta_2 r^2)^2 P^{\text{ss}}}{\Delta} r \mathrm{d}r \mathrm{d}\theta \tag{39}$$

with free energy cost per cycle

$$\Delta W = \dot{W} \times \frac{2\pi}{\langle \varOmega \rangle} = \frac{2\pi}{\Delta} \times \frac{\langle r^2(\omega + \beta_2 r^2)^2 \rangle}{\langle (\omega + \beta_2 r^2) \rangle} \tag{40}$$

Note that the integrals (averages) we are going to calculate all take the form $I_n = \int_0^\infty r^{2n} \exp[-(\beta_1 r^4/4 - \mu r^2/2)/\Delta] \mathrm{d}(r^2) = \int_0^\infty x^n \exp[-(\beta_1 x^2/4 - \mu x/2)/\Delta] \mathrm{d}x$. Making the change of variable $y = \rho(x/r_{\text{s}}^2 - 1)$, we have

$$I_n = \exp(\rho^2)(r_{\text{s}}^2/\rho)^{n+1} \int_{-\rho}^{\infty} (y + \rho)^n e^{-y^2} \mathrm{d}y \tag{41}$$

In the limit of $\rho \gg 1$, integrals $\int_{-\rho}^{\infty} p_n(y) e^{-y^2} \mathrm{d}y$ can be estimated by simple Gaussian integrals $\int_{-\infty}^{\infty} p_n(y) e^{-y^2} \mathrm{d}y$ where $p_n(y)$ are polynomials of degree n. Therefore, the leading terms in ΔW yields

$$\begin{aligned} \Delta W &\approx \frac{2\pi}{\Delta} \times \frac{\omega^2 r_{\text{s}}^2 + 2\omega\beta_2 r_{\text{s}}^4 (1 + 1/2\rho^2) + \beta_2^2 r_{\text{s}}^6 (1 + 3/2\rho^2)}{\omega + \beta_2 r_{\text{s}}^2} \\ &= 4\pi\rho^2 \Big(\frac{\beta_2}{\beta_1}\Big) \times \frac{\kappa^2 + 2\kappa(1 + 1/2\rho^2) + (1 + 3/2\rho^2)}{(1 + \kappa)} \\ &\approx 4\pi\rho^2 (1 + \kappa) \Big(\frac{\beta_2}{\beta_1}\Big) \end{aligned} \tag{42}$$

where $\kappa = \omega/\beta_2 r_{\text{s}}^2$ is another dimensionless parameter. If we assume that the radial contribution of phase sensitivity is much greater, i.e., $\beta_2/\beta_1 \gg 1$, then $\chi \approx \beta_2/\beta_1$ and $\kappa \ll 1$. Inserting this into (42), the linear enhancement of χ by energy dissipation emerges:

$$\chi \approx K_W \Delta W \tag{43}$$

where $K_W \approx [4\pi\rho^2]^{-1}$ is a β_2-independent constant.

Supplementary Note 9. A Toy Model to Understand the Relation Between Phase Sensitivity and Energy Dissipation

To gain more insights into the microscopic mechanism of how free energy is used to enhance phase sensitivity, we developed a simplified theoretical model of limit cycle oscillators. As shown in Supplementary Figure 6, the inner ring represents the limit cycle with an amplitude (radius) r_{s} and the outer ring represents the perturbed states with an amplitude change Δr. There are a total of $2 \times N$ discrete states for both the inner and the outer cycles. The equilibrium transition rates between adjacent states (both forward and backward) are given by $k_{\text{in}}, k_{\text{out}}$, and k_{jump}. In the nonequilbrium model, we consider the simple case where all forward rates (the thick arrows in Supplementary Figure 6) are regulated by the same nonequilibrium factor $1/\gamma_+$; similarly backward rates by γ_-. Note that even though $\gamma_\pm$ are related to $\gamma_{1,2}$ used before, they are not the same. These “microscopic” nonequilibrium factors $\gamma_\pm$ act on all transition rates in the toy model whereas $\gamma_{1,2}$ only acts on specific reactions in a realistic biochemical network.

The toy model has rotation symmetry in phase space, the steady state probability is evenly distributed at each phase $\phi_i = 2\pi i/N (i = 1, \ldots, N)$, i.e., $P_{\text{in}}^{\text{ss}}(\phi_i) + P_{\text{out}}^{\text{ss}}(\phi_i) = 1/N$ with $P_{\text{in}}^{\text{ss}}(\phi_i) \gg P_{\text{out}}^{\text{ss}}(\phi_i)$. The energy dissipation rate are dominated by the inner cycle:

$$\dot{W} \approx -(1/\gamma_+ - \gamma_-) k_{\text{in}} \ln(\gamma_+ \gamma_-). \tag{44}$$

When a perturbation moves the system to the outer cycle, a phase shift $\Delta\phi$ results from the difference of angular speeds $\Delta\omega = \frac{2\pi}{N}(1/\gamma_+ - \gamma_-)\Delta k$ between the two cycles with $\Delta k \equiv k_{\text{out}} - k_{\text{in}}$. Assuming the time to jump back to the inner cycle is τ_{jump}, we have $\Delta\phi = \Delta\omega \times \tau_{\text{jump}}$. The phase sensitivity, defined as the dimensionless phase gradient, can thus be obtained:

$$\chi = \frac{\Delta\phi}{\Delta r/r_{\text{s}}} = \frac{1-\gamma_+\gamma_-}{1+\gamma_+\gamma_-}\chi_0, \tag{45}$$

where $\Delta r/r_{\text{s}}$ represents the relative deviation from limit cycle and τ_{jump} is determined by $\tau_{\text{jump}}^{-1} = (k_{\text{jump}}^+ + k_{\text{jump}}^-) = k_{\text{jump}}(1/\gamma_+ + \gamma_-)$. The parameter $\chi_0 \equiv \frac{2\pi}{N} \times \frac{\Delta k}{k_{\text{jump}}}/\frac{\Delta r}{r_{\text{s}}}$ is a sensitivity constant depending only on equilibrium properties of the system.

From Supplementary Equation (44), the total energy dissipated per period is $\Delta W = \dot{W} \times \tau \approx -N\ln(\gamma_+\gamma_-)$. Together with Supplementary Equation (45), we have

$$\chi = \chi_0 \frac{e^{\Delta W/N}-1}{e^{\Delta W/N}+1} \approx K_W \Delta W, \quad \text{when } \Delta W \ll N \tag{46}$$

with $K_W = \frac{\chi_0}{2N}$. The equation agrees with main text Eq. (6) from direct simulations and main text Eq. (16) from analytical results.

In this simple model, the phenomenological phase-amplitude coupling parameter β_2 introduced in SLE can be calculated explicitly:

$$\beta_2 = \frac{\Delta\omega}{\Delta(r^2)} = (2r_{\text{s}})^{-1}\frac{\Delta\omega}{\Delta r} = \frac{\pi(\gamma_+^{-1} - \gamma_-)\Delta k}{N r_{\text{s}} \Delta r}, \tag{47}$$

which clearly indicates that free energy cost of increasing forward rates and suppressing backward rates both strengthen the phase-amplitude coupling β_2, therefore enhancing the phase sensitivity.

Supplementary Note 10. Design Principles for Phase Sensitivity

All biochemical oscillation systems must have (1) at least one dissipative (nonequilibrium) reaction loop, and (2) at least two different stoichiometric change vector directions in the phase space (no oscillation on a 1D chain). Here we consider a minimal model in the phase space with only two possible "jumping" directions $\boldsymbol{v}_0, \boldsymbol{v}_1$ and $\boldsymbol{v}_1$ being the dissipative link. Denote $\cos\theta = \langle \boldsymbol{v}_0, \boldsymbol{v}_1 \rangle$. See Supplementary Figure 14 for details.

Specifically, the reactions that form the dissipative loop (like the one shown in main text Fig. 2c) are assumed to have total transition fluxes w_1 along the direction $\boldsymbol{v}_1$. In addition, we also include the reaction (along $\boldsymbol{v}_0$) that is not directly driven by chemical force, e.g., the reaction between X and A in the Brusselator model. The net flux for this non-dissipative reaction is denoted by w_0.

To look for strategy in enhancing phase sensitivity of limit cycle oscillation, we calculate χ by projecting the transition fluxes w (along) in the state space onto the phase (angular) direction and the amplitude (radial) directions.

We can compute

$$|\nabla_{\boldsymbol{x}^*}\phi| = \frac{\Delta w_\parallel \tau_{\text{jump}} \times (2\pi/r_{\text{s}})}{\Delta r/r_{\text{s}}} = 2\pi \frac{\Delta w_\parallel/(\Delta r/r_{\text{s}})}{\Delta w_\perp/(\Delta r/r_{\text{s}})} \tag{48}$$

where $w_\parallel$ and $w_\perp$ are the rates along and perpendicular to the limit cycle; $\tau_{\text{jump}} = \Delta r/\Delta w_\perp$. The change of transition rates Δw are induced by a putative perturbation causing a relative change $\Delta r/r_{\text{s}}$ of amplitude. Note that the progression direction $\boldsymbol{v}_\parallel$ is determined by both dissipative and non-dissipative reactions. Let $\theta_{0,1}$ be the angle between $\boldsymbol{v}_{0,1}$ and $\boldsymbol{v}_\parallel$. The direction of $\Delta w_{0,1}$ are chosen such that $\Delta w_\perp$ generates a jump process approaching the limit cycle. Using the geometric relationship of different transition rate vectors, we can rewrite $\Delta w_{\perp,\,\parallel}$ as $\Delta w_\parallel = \Delta w_1 \cos\theta_1 - \Delta w_0 \cos\theta_0$ and $\Delta w_\perp = \Delta w_1 \sin\theta_1 + \Delta w_0 \sin\theta_0$. Plugging these expressions into Supplementary

Equation (48), we have

$$|\nabla_{\boldsymbol{x}^*}\phi| = \frac{\Delta w_1(w_1 + w_0\cos\theta) - \Delta w_0(w_0 + w_1\cos\theta)}{(w_1\Delta w_0 + w_0\Delta w_1)\sin\theta}$$
$$= \frac{k_q(k_w + \cos\theta) - (1 + k_w\cos\theta)}{(k_w + k_q)\sin\theta} \tag{49}$$

where $k_w = w_1/w_0$ and $k_q = \Delta w_1/\Delta w_0$. In our model, we use w_0 as a reference rate that doesn't vary much with γ.

Next, we analyze the introduced dimensionless variables to search for the factors that affect phase sensitivity. The ratio k_q can be rewritten in the form

$$k_q = \frac{w_f}{w_0} \times \frac{|q_f - (w_b/w_f)q_b|}{q_0} \equiv c_w\frac{|q_f - r_w q_b|}{q_0} \tag{50}$$

where $w_{f,b}$ is the net forward/backward flux along phase progression direction. $q_i = \frac{\Delta w_i/w_i}{\Delta r/r_s}$ is the relative change of transition rates induced by a perturbation given by a relative change $\Delta r/r_s$ of the amplitude. q_i characterizes the sensitivity the transition determined by the nonlinearity in the underlying reaction rates. For example, reactions of (pseudo) first order have $q_i \sim 1$; the autocatalytic reactions in the Brusselator model give $q_i \sim 3$.

By definition, the ratio of backward to forward flux r_w varies between 0 and 1 along the circle. Also, we would expect the factor $c_w = w_f/w_0 \gg 1$ since it is, in general, greatly enhanced by reducing γ. Now using these two parameters, k_w can be expressed as $c_w(1-r_w)$. Substituting these two expressions into Supplementary Equation (49), we have $|\nabla_{\boldsymbol{x}^*}\phi| = F(c_w, r_w)$ and $\chi = \max_{\arg\ r_w} F(c_w, r_w)$. The function F takes the form

$$F(c_w, r_w) = \left|\frac{c_w|q_f - r_w q_b|(c_w(1-r_w) + \cos\theta) - (1 + c_w(1-r_w)\cos\theta)q_0}{\sin\theta[(1-r_w)q_0 + |q_f - r_w q_b|]c_w}\right| \tag{51}$$

Note that c_w^2 term dominates the numerator while c_w leads the denominator. Thus for $0 < r_w \leq 1$,

$$F(c_w, r_w) \sim c_w \csc\theta[q_0|q_f - r_w q_b|^{-1} + (1-r_w)^{-1}]^{-1}, \tag{52}$$

which is a decreasing function of r_w. The maximum is achieved when $\min r_w \to 0$. Equation (52) clearly indicates that there are two major factors at play:

1. When dissipation increases, the general growing trend of χ is credited to c_w, i.e., the overall enhancement of global flux by decreasing γ;
2. At a fixed dissipation, the net flux ratio r_w further fine tune the sensitivity; higher sensitivity is favored by unbalanced net flux partition (small r_w).

Supplementary Note 11. Verification of the Design Principles in Kai System

We further test the design principles in a mathematical model for the *in vitro* KaiC oscillator proposed by Rust et al [1]. As shown in Supplementary Figure 10a, four KaiC phosphorylation states – unphosphorylated KaiC (U), T432-phosphorylated KaiC (T), S431-phosphorylated KaiC (S), and double-phosphorylated KaiC (D) – form the nonequilibrium loop. The (de)phosphorylation of different phosphorylated forms of KaiC depends on the global KaiA activity, which is in term controlled by KaiB:

$$k_p = \frac{1}{1 + r_p K_{\text{rel}}} \times \frac{k^A_{\text{phos}}[A]}{K_{1/2} + [A]} \tag{53}$$

$$k_{dp} = k^0_{\text{dphos}} + \frac{k^A_{\text{dphos}}[A]}{K_{1/2} + [A]} \tag{54}$$

where r_p is the [ADP]/[ATP] ratio and $[A]$ is the concentration of free KaiA. For each of the (de)phosphorylation processes, there is a basal rate constant in the absence of KaiA (k^0_{dphos}), a rate constant describing the maximal effect of KaiA ($k^A_{\text{(d)phos}}$) and a constant $K_{1/2}$ describing the concentration of KaiA needed to produce a half-maximal effect on KaiCs activity. Apart from the K_{rel} parameter which describes the relative affinity of ATP and ADP, all

parameters use the values set previously by experimental data (see Table S1 and [1, 6] for details). Note that k^{A}_{phos} and $k^{0,A}_{\text{dphos}}$ are different for each phosphorylation/dephosphorylation link, and were separately measured [1, 6]. We choose $K_{\text{rel}} = 1.0$ in our simulation (same value in [1]) and set r_p to 0 to mimic the experimental condition in [1] (100% ATP).

The kinetic equations of the model are [1]:

$$\begin{aligned}\frac{d[T]}{dt} &= k_{U\to T}[U] + k_{D\to T}[D] - k_{T\to D}[T] - k_{T\to U}[T]\\ &\equiv J_{U\to T} + J_{D\to T} - J_{T\to D} - J_{T\to U} \qquad (55)\\ \frac{d[D]}{dt} &= k_{T\to D}[T] + k_{S\to D}[S] - k_{D\to S}[D] - k_{D\to T}[D]\\ &\equiv J_{T\to D} + J_{S\to D} - J_{D\to S} - J_{D\to T} \qquad (56)\\ \frac{d[S]}{dt} &= k_{U\to S}[U] + k_{D\to S}[D] - k_{S\to D}[S] - k_{S\to U}[S]\\ &\equiv J_{U\to S} + J_{D\to S} - J_{S\to D} - J_{S\to U} \qquad (57)\\ [U] &= [C]_{\text{total}} - [T] - [D] - [S] \qquad (58)\\ [A] &= \max\{0, [A]_{\text{total}} - 2[S]\} \qquad (59)\end{aligned}$$

where $[C]_{\text{total}}$ and $[A]_{\text{total}}$ are the total concentration of KaiC and KaiA, and all the Js represent the chemical fluxes. Each $k_{X\to Y}$ is computed from Supplementary Equation (53 & 54) depending on the type of $X \to Y$ transition (phosphorylation or dephosphorylation step). It is assumed here that S-state KaiC in complex with KaiB rapidly sequesters KaiA with extremely high affinity (Supplementary Equation (59) above) [1].

Since not all the microscopic reverse reaction rates were measured experimentally, we test the first principle of suppressing phase diffusion on the whole $U \to T \to D \to S \to U$ cycle for an individual KaiC molecule. Instead of having two states ("X" and "Y") forming the cycle with two antiparallel pathways as in the case of the Brusselator model, there are 4 states (U, T, D, S) with 4 links (pathways) between them in the KaiC phosphorylation cycle as there are two phosphorylation sites (S and T) for a KaiC molecule. We calculate the ratio of forward to backward flux $r_{X\leftrightarrows Y} = J_{X\to Y}/J_{Y\to X}$ on each link $X \leftrightarrows Y$. Note that the direction of forward and backward directions $X \leftrightarrows Y$ is chosen along the $U \to T \to D \to S \to U$ cycle in accordance with the notation of parallel and antiparallel pathways used in our theory. As we did in the Brusselator model, we compare the period-averaged ratio $\langle r_{X\leftrightarrows Y}\rangle_\tau$ to see whether this ratio for different links along the cycle are balanced. Using the experimentally determined parameters, the KaiC model yields

$$\langle r_{U\leftrightarrows T}\rangle_\tau = 1.10, \quad \langle r_{T\leftrightarrows D}\rangle_\tau = 1.25, \quad \langle r_{D\leftrightarrows S}\rangle_\tau = 1.07, \quad \langle r_{S\leftrightarrows U}\rangle_\tau = 1.14. \qquad (60)$$

This result strongly indicates that the balanced dissipative cycle of KaiC oscillation is properly designed to minimize phase diffusion and enhance timing accuracy.

For the second principle of phase sensitivity, we calculate the net flux for each link $J^{(\text{net})}_{X\to Y} = J_{X\to Y} - J_{Y\to X}$. The net phosphorylation and dephosphorylation flux are then approximated as $J^{(\text{net})}_{U\to D} = J^{(\text{net})}_{U\to T} + J^{(\text{net})}_{T\to D}$ and $J^{(\text{net})}_{D\to U} = J^{(\text{net})}_{D\to S} + J^{(\text{net})}_{S\to U}$ respectively. As shown in Supplementary Figure 10b, the backward-to-forward (in terms of phosphorylation rhythm) net flux ratio r_w is smaller during the subjective day than the night, indicating a higher phase sensitivity during the phosphorylation phase. This argument is supported by the experimental PRC of *in vitro* Kai system reported in [1] (shown in Supplementary Figure 10a). In our simulation the average r_w during the subjective day and night are $\langle r_w\rangle_{\text{day}} \approx 0.2$ and $\langle r_w\rangle_{\text{night}} \approx 0.5$. This result suggests that the Kai system differentially distributes phosphorylation and dephosphorylation net flux to achieve desirable phase sensitivity.

Supplementary Note 12. Nonequilibrium Phase FRR

We now apply the same procedure as in (10) to noisy SLE (29), and focus on its phase dynamics. In this case, $\Omega = \omega + \beta_2 r_s^2$, and the noise term takes the form $g(\phi,t) = (-\frac{\beta_2}{\beta_1}/r)\eta_r + (1/r)\eta_\theta$, where $\eta_r = \eta_1\cos\theta + \eta_2\sin\theta$ and

$\eta_\theta = -\eta_1 \sin\theta + \eta_2 \cos\theta$. Particularly, we obtain

$$\begin{aligned}\langle g(\phi,t)g(\phi,t')\rangle &\equiv D(\phi)\delta(t-t') \\ &= \frac{1}{r^2V}\left[\Big(\frac{\beta_2}{\beta_1}\Big)^2 Q_{rr} - 2\Big(\frac{\beta_2}{\beta_1}\Big)Q_{r\theta} + Q_{\theta\theta}\right]\delta(t-t')\end{aligned} \tag{61}$$

with

$$\begin{aligned}Q_{rr} &= Q + \frac{1}{2}(Q_{11}-Q_{22})\cos 2\theta + Q_{12}\sin 2\theta \\ Q_{\theta\theta} &= Q + \frac{1}{2}(Q_{22}-Q_{11})\cos 2\theta - Q_{12}\sin 2\theta \\ Q_{r\theta} &= \frac{1}{2}(Q_{22}-Q_{11})\sin 2\theta + Q_{12}\cos 2\theta\end{aligned} \tag{62}$$

where $Q = (Q_{11}+Q_{22})/2$.

Using the stochastic average method, we can calculate

$$D_\phi = \frac{QV^{-1}}{r_{\mathrm{s}}^2}(\beta_2^2/\beta_1^2 + 1) \tag{63}$$

It is straightforward from (63) and main text Eq. (13) that the dimensionless phase diffusion constant is related to phase sensitivity in the SLE model by

$$D = D_\phi \times T = \frac{QV^{-1}\tau}{r_{\mathrm{s}}^2} \times \chi^2 \tag{64}$$

This establishes a relation between phase diffusion constant D and sensitivity χ in a form very similar to the FRR. It's then natural to introduce an "effective temperature" $T_{\mathrm{eff}} = D/\chi^2$. In the regime near bifurcation, the physical meaning of $T_{\mathrm{eff}} = QV^{-1}\tau/r_{\mathrm{s}}^2$ can be interpreted as the relative amplitude fluctuations of oscillating species accumulated during a cycle. For specific biochemical reactions, the foregoing analyses indicate that QV^{-1} is just some linear combinations of period averaged Poisson noise arising from various reaction channels.

Unlike the equilibrium FRR, where the linear dependence is independent of other parameters, this phase FRR is not unique. As seen from the expression (42) of dissipation ΔW, there are many degrees of freedom we can vary in the high dimensional parameter space to break detailed balance, upon which both T_{eff} and the relation between χ and D depend. Three different situations are listed as follows:

1. μ is varied while other parameters are fixed: $T_{\mathrm{eff}} \sim \Delta W^{-1}, \chi = \text{const.}$, and hence $D \sim \Delta W^{-1}$;
2. β_2 is varied while other parameters are fixed: $T_{\mathrm{eff}} = \text{const.}, \chi \sim \Delta W$, but at the cost of $D \sim \chi^2 \sim \Delta W^2$;
3. μ and β_2 are jointly regulated with constraint that $\mu \propto \beta_2$: $T_{\mathrm{eff}} \sim \Delta W^{-1/3}, \chi \sim \Delta W^{1/3}$, and $D \sim \Delta W^{1/3} \propto \chi$.

Indeed, the phase sensitivity can have positive correlation as well as negative one when changing parameters along a certain direction. Nevertheless, if the direction is properly chosen, we can keep the best of both world, i.e., having a high sensitivity and yet a small fluctuation. The biochemical oscillators fueled by nonequilibrium reaction loops, for example, can generate a complex dependence like (30) on energy parameter γ.

As illustrated in the simple model shown in Supplementary Figure 6, our study revealed three tightly related effects of free-energy dissipation in oscillatory systems: (1) Energy cost can constrain the probability distribution near the stable limit cycle, i.e., significantly lowers the "effective temperature" for fluctuations around the limit cycle (see Supplementary Figure 15). Consequently, even if the system is subjected to phase FRR, coherent oscillation and phase responsiveness is compatible; (2) Extra energy dissipation is required for noisy oscillators to suppress phase diffusion as shown here and in our previous work [2]; (3) Free energy consumption can be used to enhance the phase-amplitude coupling, which in turn enhances the phase sensitivity to external signals/perturbations.

Supplementary References

[1] Rust, M. J., Golden, S. S. & O'Shea, E. K. Light-driven changes in energy metabolism directly entrain the cyanobacterial circadian oscillator. *Science* **331**, 220–223 (2011).
[2] Cao, Y., Wang, H., Ouyang, Q. & Tu, Y. The free-energy cost of accurate biochemical oscillations. *Nat. Phys.* **11**, 772–778 (2015).
[3] Nakashima, H. & Feldman, J. F. Temperature-sensitivity of light-induced phase shifting of the circadian clock of neurospora. *Photochem. Photobiol.* **32**, 247–251 (1980).
[4] Broda, H., Johnson, C. H., Taylor, W. R. & Hastings, J. Temperature dependence of phase response curves for drug-induced phase shifts. *J. Biol. Rhythms* **4**, 327–333 (1989).
[5] Barrett, R. & Takahashi, J. Lability of circadian pacemaker amplitude in chick pineal cells: A temperature-dependent process. *J. Biol. Rhythms* **12**, 309–318 (1997).
[6] Rust, M. J., Markson, J. S., Lane, W. S., Fisher, D. S. & O'shea, E. K. Ordered phosphorylation governs oscillation of a three-protein circadian clock. *Science* **318**, 809–812 (2007).
[7] Kuramoto, Y. *Chemical Oscillations, Waves and Turbulence*, vol. 19 of *Springer Series in Synergetics* (Springer, 1984).
[8] Goldobin, D. S., nosuke Teramae, J., Nakao, H. & Ermentrout, G. B. Dynamics of limit-cycle oscillators subject to general noise. *Phys. Rev. Lett.* **105**, 154101 (2010).
[9] Gillespie, D. T. The chemical langevin equation. *J. Chem. Phys.* **113**, 297–306 (2000).
[10] Taylor, S. R., Gunawan, R., Petzold, L. R. & Doyle, F. J. Sensitivity measures for oscillating systems: Application to mammalian circadian gene network. *IEEE T. Automat. Contr.* **53**, 177–188 (2008).
[11] Barato, A. C. & Seifert, U. Coherence of biochemical oscillations is bounded by driving force and network topology. *Phys. Rev. E* **95**, 062409 (2017).
[12] Gillespie, D. T. Exact stochastic simulation of coupled chemical reactions. *J. Chem. Phys.* **81**, 2340–2361 (1977).
[13] Kuznetsov, Y. A. *Elements of Applied Bifurcation Theory*, vol. 112 of *Applied Mathematical Sciences* (Springer, 1998).
[14] Roberts, J. & Spanos, P. Stochastic averaging: An approximate method of solving random vibration problems. *Int. J. Non-Linear Mechanics* **21**, 111–134 (1986).
